# Arrays Formation of Zinc Oxide Nano-Objects with Varying Morphology for Sensor Applications

**DOI:** 10.3390/s20195575

**Published:** 2020-09-29

**Authors:** Serguei P. Murzin, Nikolay L. Kazanskiy

**Affiliations:** 1 Samara National Research University, Moskovskoe Shosse 34, 443086 Samara, Russia; kazanskiy@ipsiras.ru; 2 Institute of Production Engineering and Photonic Technologies, TU Wien, Getreidemarkt 9, 1060 Vienna, Austria; 3 Image Processing Systems Institute of RAS—Branch of the Federal Scientific Research Centre Crystallography and Photonics of Russian Academy of Sciences, Molodogvardejskaya street 151, 443001 Samara, Russia

**Keywords:** laser treatment, heat exposure, sound waves induced by laser, formation, nano-object, ZnO nanowires, diffraction grating, sensor

## Abstract

The regularities and features of the formation of arrays of zinc oxide nano-objects with varying morphology are determined by CO_2_ laser processing with intensification of diffusion processes in the solid state of Cu–Zn metallic materials which are selectively oxidizable. In the process of laser treatment in air using the synergy of heat exposure and vibrations induced by laser with a force fundamental frequency of 100 Hz, the brass surface of samples is oxidized mainly with the generation of ZnO nanowires. The condition for intensification is the local non-stationary deformation caused by sound waves induced by laser. Upon the initiation of the processes of exfoliation of the initially formed layers on the material surface, apart from a disordered structure, a structure was formed in the central region containing two-dimensional objects made of zinc oxide with characteristic thicknesses of 70–100 nm. Such arrays can provide the potential to create a periodic localized electric field applying direct current, this allows the production of electrically switched diffraction gratings with a variable nature of zones. It has been established that during laser pulse-periodic irradiation on brass, the component of the metal alloy, namely, zinc, will oxidize on the surface in the extent that its diffusion to the surface will be ensured. During laser pulse-periodic heating under conditions of the experiment, the diffusion coefficient was 2–3 times higher than from direct heating and exposure to a temperature of 700 °C. The study of the electrical resistance of the created samples by the contact probe method was performed by the four-point probe method. It was determined that the specific electrical resistance at the center of the sample was 30–40% more than at the periphery. To determine the possibility of using the obtained material based on zinc oxide for the creation of sensors, oxygen was adsorbed on the sample in an oxygen–argon mixture, and then the electrical resistance in the central part was measured. It was found that the adsorbed oxygen increases the electrical resistivity of the sample by 70%. The formation of an oxide layer directly from the metal substrate can solve problem of forming an electrical contact between the gas-sensitive oxide layer and this substrate.

## 1. Introduction

Diffraction gratings are optical components that can split the incident beam and are used in optical tasks in which it is difficult to realize the required intensity function using refractive or reflective optics [1]. Due to such opportunities, these are widely used in many optical devices, for example, for constructing sensors of angular and linear displacements, as well as deformation sensors, etc. [2]. Electrically switchable diffraction gratings, which operate under the control of the applied electrical energy find application [3,4]. In Reference [5] a hybrid liquid crystal and carbon nanotube-based device was demonstrated, which showed the ability to function as a switchable diffraction grating. When implementing such a system, one among the most important stages is the manufacture of either a periodic (with a period, comparable to the wavelength of the incident beam), or a gradient matrix of electrodes that can generate a local controlled electric field. Reference [6] showed that a nanorod or nanowire ZnO structure can create a periodic localized electric field under applied direct current which can modulate the optical properties of the medium in which liquid crystals are dissolved. Vertically aligned ZnO nanostructure elements were synthesized by hydrothermal growth on a two-dimensional layer.

Zinc oxide is a semiconductor compound that with its piezo and ferroelectric properties due to its practical and promising potential applications, is increasingly coming to the researchers’ attention [7,8,9]. Special attention is devoted to the generation of structures that are based on nanoelements [10,11,12,13,14] (nanofilms, nanofibers, nanowires, nanorods, etc.). These type of structures can be applied in sensor devices, which in this case obtain explicit advantages compared to sensors available on the market [15,16,17,18]. For example, sensors that are based on nanoelements provide an increase in the selectivity and a lower energy consumption. In addition to the manufacturing of gas and liquid sensors, ZnO is increasingly applied for ultraviolet lasers and Light-emitting diodes production, as well as for the manufacture of piezoelectric devices, scintillators, solar cells, and others [19,20,21,22,23]. Among the feasible technical implementations of these structures are metal/oxide layered materials that present great interest as functional electro-contact materials.

In References [24,25], it was also stated that arrays of ZnO nanorods or nanowires vertically oriented to the substrate can be produced using the direct current electrochemical method, but it was noted that ZnO morphology is extremely sensitive to synthesis conditions. In Reference [26], it was shown that for two-dimensional (2D) diffraction gratings, it is effective to realize zones different from periodic slit- and ring-like ones, this allows the realization of new functional capabilities of diffractive optical elements. It was noted that despite the apparent obviousness since the creation of the zone plate, this issue has not been raised for many years due to the lack of practical possibility of realizing zone plates with a variable nature of zones. The necessary change is preferably not only in space, but also in time. In this regard, the application of nanostructured arrays based on ZnO with a smoothly changing morphology depending on plane coordinates is promising. These arrays are capable of creating a localized electric field.

Despite the fact that zinc oxide is not a new material and has been studied for many decades, interest in studying and obtaining materials structures with a zinc oxide base, including at nanoscale, has increased significantly in recent years [27,28,29]. Thin layers of zinc oxide are created by the application of physical and chemical methods (chemical vapor-phase deposition, pulsed laser ablation, electro-deposition, thermal evaporation, etc.). Nowadays obtaining structures that are based on nanoelements is of significant attention, because it allows the researchers to expand functional abilities of the existing devices and create new ones. ZnO structures with high values of the specific surface area are particularly relevant. Among the practicable technical applications of such forms are metal/oxide layered materials, and for instance Cu/ZnO, which are used as functional electro-contact materials. Operational properties of such materials, as a rule, improve with an increase in the dispersity and uniformity of the oxide phase distribution on the metallic surface. For a new generation of gas-sensitive sensors, methods for obtaining layered nanomaterials with a high surface area are of great demand in the production industry.

To obtain semiconductor nanomaterials, the most widely used technologies are chemical deposition from the gas phase [30] and molecular beam epitaxy [31], which were developed originally for creation of thin-film elements in microelectronics products. Conducted research has demonstrated that the heterogeneous polycrystalline microstructure, consisting of micrograins of ZnO, can be obtained using the methods of spray coating and subsequent sintering. In particular, mixed films of oxides of copper and zinc were obtained by preliminary magnetron sputtering of zinc and copper on substrates from glass and their successive annealing in atmosphere [32,33]. A process for obtaining such structures comprises sequential steps of forming of layers of zinc and copper oxides.

However, there is currently no efficient method available by which a controlled industrial generation of nanomaterials that are ZnO-based periodic structures is feasible. Methods of nanolithography, such as photolithography and electron-beam lithography, are used to implement the process of creation of structures from nanowires and nanotubes with vertical or lateral alignment and controlled positioning [34,35,36,37]. However, typical drawbacks such as low value of productivity, small working area, and cost-intensive equipment limit their scope of application. Applied aspects of the use of ZnO-based nanomaterials are determined by not only the mechanical, optical, electrical, and structural properties of their particular elements, but also the properties associated with their morphometric parameters (the characteristic dimensions of the particular elements, the degree of dispersion, density, and uniformity of the surface distribution) [38]. For many applications, the characteristics of morphology are crucial, which is why the controlling of these parameters by modifying the synthesis conditions will be an important task but in this area many unsolved problems remain. The search for new methods for a high-performance manufacturing of nanostructures with controlled formation of periodic structures is a significant and promising direction in the field of the fundamental bases of creating functional materials.

References [39,40] evaluated possibilities for generation of nanomaterials and they synthesized nanoporous nanomaterials as well as ZnO-based composite nanomaterials using pulse-periodic laser irradiation. For the first time, a notable increase in the diffusion coefficient (several times as compared to heat exposure of the laser beam only) in the metallic material, caused by the synergy of heat exposure and vibrations induced by laser, predominantly in the range of sound frequencies, as a result of a pulse-periodic laser irradiation with a pulse duration in the milli- and microsecond range, was described. The oxide nanostructures obtained as result of thermal oxidation of metallic materials show high purity since this approach eliminates the need for chemical catalytic synthesis [41]. It should be mentioned that ZnO nanostructures were synthesized simultaneously on the substrate by directly heating brasses in air and this was described in Reference [42]. Applying pulse-periodic laser irradiation, it is possible to obtain similar nanostructures based on zinc oxide for a significant shorter time. Since the processes of ZnO formation are limited by the diffusion of zinc to the surface, this indicates an intensification of the diffusion processes, i.e., a significant increase in the diffusion coefficient in the material. The condition for intensifying diffusion processes in the solid state of Cu–Zn metallic materials which are selectively oxidizable was defined as a stress-strain non-stationary state caused by sound waves induced by laser. The use of the synergy effect under study, allows the implementation of an up to date approach for the structures generation of nanomaterials that are based on ZnO. In Reference [43], the possibility of the formation of structures of layered nanomaterials that are based on zinc oxide by laser pulse-periodic irradiation was presented. Nanostructured arrays with a morphology of ZnO nanowires varying from the center to the periphery were obtained on a current-conducting substrate from a copper–zinc alloy [44]. It was also shown in Reference [45] that when single-phase alpha-brasses (containing up to 35% Zinc) are heated in air, structures practically free of CuO are formed on the surface of the material, but only zinc oxide remains in the form of nanowires (NWs), nanoflakes (NFs), and nanopalms (NPs), depending on the heating temperature.

It is known that the effect of a significant increase in the rate of atoms movement in the solid phase of metals and alloys was identified as a new phenomenon in the study of processes occurring under mechanical shock compression [46]. The manifestation of this intensification effect of mass transfer in nonequilibrium conditions, which is caused by pulsed elastic or plastic deformation, was considered in the processes of mechanical–chemical–thermal treatment of various types: ultrasonic shock; pulsed laser; electrohydropulse; electrospark; pulsed magnetic field; deformation in the process of reversible martensitic transformations, and a number of other types of pulse loading [47,48,49]. External high-energy influences, which also include a pulse-periodic laser irradiation [50], lead to a notable increase in the atom’s mobility in alloys and metals in the solid state. Non-stationary form transformation, localized in one part of the sample, is an obligatory condition for the demonstration of a generalized thermodynamic force, providing fast mass transfer [51]. It should be noted the physics of the process of occurrence of oscillations of the sample at natural frequency and frequencies proportional to the forced fundamental frequency is described in References [52,53,54]. However, the processes physical nature that occur during formation and successive evolution of gradient structural phased states is poorly studied [55,56]. This is due to the fact that mass transfer under substantially nonequilibrium conditions is a result of simultaneous action of several processes of various physical nature, such as, a change in the state of the structure of a metal material, the development and relaxation of various crystal defects, occurrence of stresses, etc. The change in the morphology of arrays of zinc oxide nano-objects obtained by laser treatment using the synergy of heat exposure and vibrations induced by laser in the frequency range of sound remains practically unstudied. Thus, the objective of this study was to determine the regularities and features of the formation of arrays of zinc oxide nano-objects with varying morphology by laser treatment with intensification of mass transfer in the solid phase of selectively oxidized copper–zinc metal materials.

As a rule, it is possible to obtain ZnO structures relatively evenly distributed over the sample area using a direct synthesis of ZnO nanowires on brass by thermal oxidation [57]. Laser exposure only on the central part of the sample makes it possible to obtain ZnO structures which will be extremely unevenly distributed over the sample area. In References [43,44], and also in Reference [58], the effect on the material structure of laser irradiation in the sound range with a frequency of 500 Hz and in the infrasonic range with a frequency 3 Hz was studied. Considering the laser action with a frequency of 100 Hz and comparing it with these previously obtained results, it is possible to highlight the most characteristic patterns and features of the synergy of heat exposure and laser-induced vibrations in the sound frequency range, including an eventual effect of the different laser frequency on the nanowires growth.

## 2. Materials and Methods

**Figure 1:**
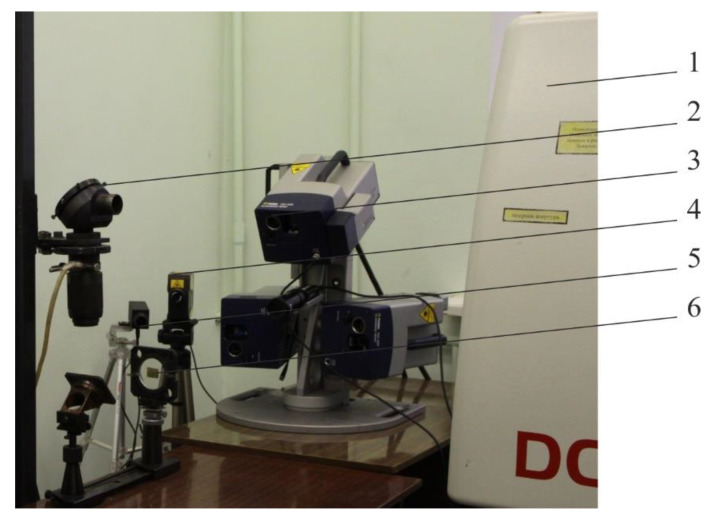
Photo of an experimental setup for samples processing and study of vibrational characteristics of objects during the formation of arrays of zinc oxide nano-objects: 1—ROFIN DC 010 radio frequency excited and diffusion-cooled CO_2_ laser; 2—a mirror of the optical system; 3—Polytec PSV-400-3D three-axis scanning laser vibrometer; 4—Polytec PDV 100 two-coordinate measuring device (Polytec GmbH, Waldbronn, Germany); 5—Kelvin LCM-1300M non-contact thermometer (Astena, Ryazan, Russia); 6—sample from brass L62.

Samples from Cu–Zn brass alloy L62 with dimensions 30 × 20 × 0.05 mm were processed. For processing, a ROFIN DC 010 CO_2_ laser (ROFIN-SINAR Laser GmbH, Hamburg, Germany) with diffusion cooling and radio frequency excitation with a duration of a single pulse of 0.026–125 ms was used. The method of laser scanning vibrometry was applied, as a modern method for measuring the parameters of mechanical vibrations of samples. Advantages of this method are the possibility of remote non-contact vibration measurement in the absence of influence on resonance properties of samples, as well as the possibility of measurements without preliminary preparation of the sample surface and the rapid measurement of vibrations at various points of the sample. When using this method, the vibrations of the reflecting surface modulate the frequency shift, and the electronic processing of this modulation signal makes it possible to determine the vibration parameters. In contrast to contact measuring methods, the test sample is unaffected by the vibration measuring process. Other advantages of optical vibrometers are their high accuracy and measurement speed. The vibration rate was measured using a Polytec PSV-400-3D three-components scanning laser vibrometer (Polytec GmbH, Waldbronn, Germany). Figure 1 shows an experimental setup for samples processing and study of vibrational characteristics of objects during the formation of arrays of zinc oxide nano-objects on the Cu–Zn alloy surface.

Macroscopic analysis was performed in order to get an idea about the general structure, and to select those areas that require further microscopic examination. Microanalysis allowed the characterization of the size and location of components present in the material. The microstructure was studied using a Neophot-30 metallographic microscope (Carl Zeiss Jena GmbH, Jena, Germany). The scanning electron microscopy (SEM) that is designed to obtain an image of the sample surface with a high spatial resolution, as well as information on the composition, structure, and some other properties of subsurface layers was applied. In SEM mode of detection of secondary electrons, images displaying the topography of the surfaces were created. It became possible to study the fine structure of the material, since the SEM magnification limit is significantly greater than the best light microscopes. SEM images of the surface were obtained using a Quanta microscope (FEI Company, Hillsboro, Oregon, USA). X-ray spectroscopy allowed the determination of elemental chemical composition of samples. An analysis of the elemental chemical composition was performed by the method of energy-dispersive X-ray spectroscopy (EDXS) using a VEGA TESCAN SEM microscope (TESCAN ORSAY HOLDING, a.s., Brno, Czech Republic).

X-ray spectral analysis was performed using an REM-100U scanning electron microscope (JSC "SELMI", Sumy, Ukraine). To perform an X-ray diffraction analysis, was used an DR-01 Radian X-ray diffractometer (Eksperttsentr, Moscow, Russia). A computer-aided resistivity meter ROMETER was used to assess the local specific electrical resistance of different surface areas. It had the following parameters: measurement range of resistivity, Ohm × cm: from 0.001 to 10^4^, limits of allowable relative measurement were not more than error ±2% for resistivity within the range 0.011–10^4^ Ohm × cm.

## 3. Results and Discussion

**Figure 2:**
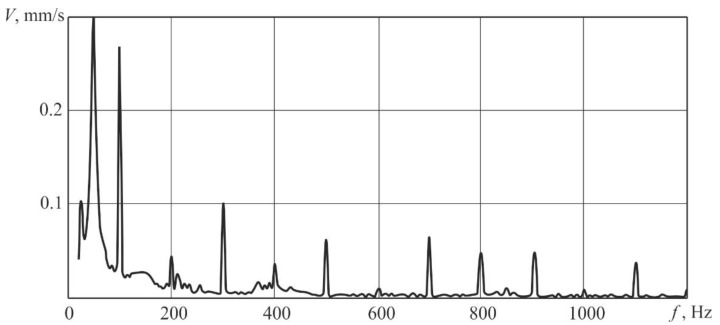
The spectrum of vibration rate averaged over the samples surface under laser irradiation with a pulse frequency f = 100 Hz.

Pulse-periodic laser irradiation was performed with a frequency of 100 Hz, the average laser beam power was 330 W. The spectra of samples’ responses to vibrational excitation caused by a pulse-periodic laser irradiation were measured. A typical spectrum of the vibration rate V averaged over the samples surface is shown in Figure 2. After the results analysis of the responses of samples to the defined laser vibrational excitation, it was confirmed that the values of rate of vibration have local maxima in case of frequencies which are multiples to the force fundamental frequency, values of which decrease with increasing frequency.

**Figure 3:**
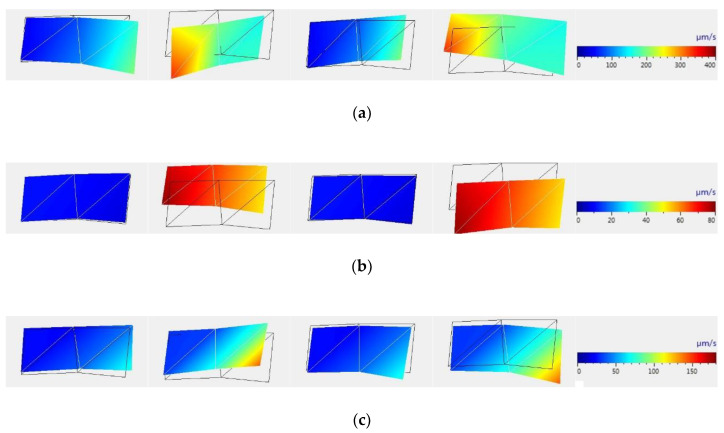
Images of samples and graphical displays of the magnitude of the vibration rate at each point of the sample at time intervals of a quarter of the oscillation cycle, obtained using PSV Presentation software that correspond to the frequencies of the sound range: 100 Hz (**a**), 200 Hz, (**b**) and 300 Hz (**c**).

Additionally, increased values of rate of vibration occur at frequencies that are near the natural oscillation frequency that for the selected sample size was calculated according to References [59,60] and was approximately 48.5 Hz. In this case, laser irradiation with pulsed-periodic beam allows the formation of a stress state in samples. Vibrational patterns of samples were determined and recorded in the case of frequencies which are multiples to the force fundamental frequency. Figure 3 shows images of samples and graphical displays of the magnitude of the vibration rate at each point of the sample at time intervals of a quarter of the oscillation cycle, which were reconstructed using PSV Presentation software. These correspond to the frequencies of the sound range: the laser frequency of 100 Hz, as well as multiple frequencies, namely 200 Hz and 300 Hz. It was noted that the maximum vibration rates at the periphery of the sample, which correspond to the laser frequency of 100 Hz as well as at multiple frequencies exceed the maximum vibration rate in the center by 15–120%.

**Figure 4:**
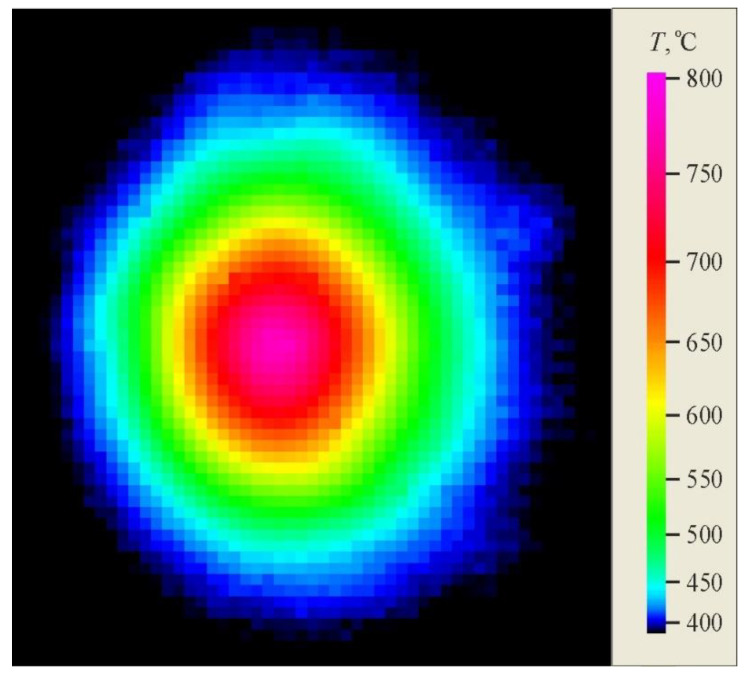
Temperature field along the sample during pulse-periodic laser treatment, exposure time 23 s.

**Figure 5:**
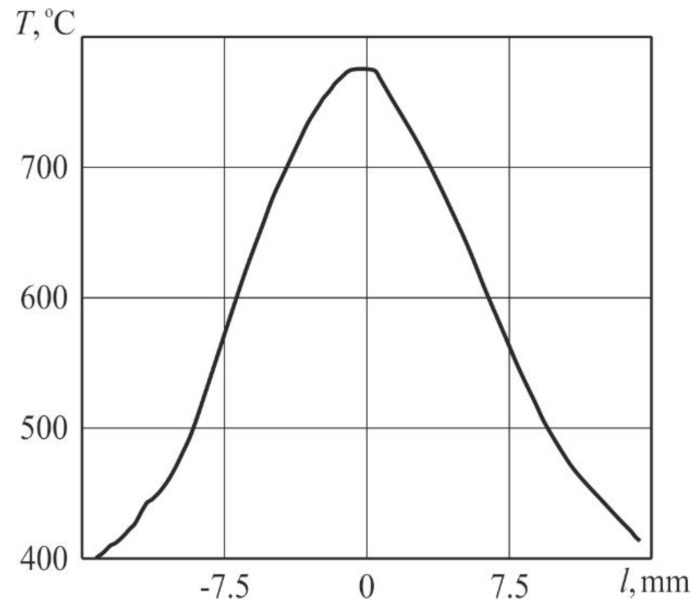
Distribution of temperature along the sample.

The temperature was recorded on the reverse surface of samples during heating by laser beam using a FLIR SC7300 thermal imaging camera (FLIR Systems AB, Täby, Sweden). It was noted that laser irradiation led to an increase in temperature, and in the central region it increased more with time than at the periphery. Figure 4 displays the temperature field in the sample and Figure 5 presents the distribution of temperature along the sample in the central region of the heat-affected zone for a pulse-periodic laser irradiation time of 23 s.

**Figure 6:**
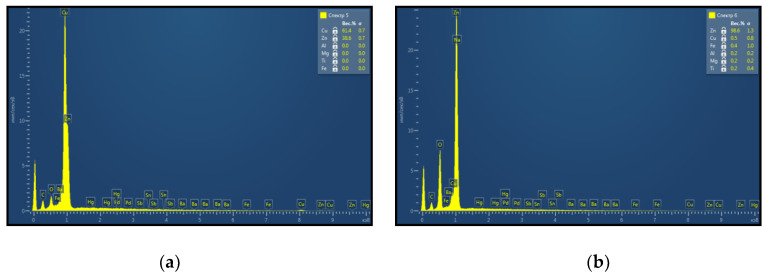
The results of the analysis of the elemental chemical composition: of the initial structure of brass (**a**) and of the surface of the treated material (**b**) after a pulse-periodic laser irradiation.

Material surface was studied after a pulsed-periodic laser treatment that was implemented in air. In this case, the formation of a oxide coating was observed on the brass surface, consisting of needle-shaped elongated crystals and having a lemon yellow colour, which, after an increasing treatment time, turned into a whitish-gray colour, which is typical for zinc oxide. An elemental composition analysis of the whitish-gray film formed on the material surface as a result of laser irradiation was made. The analysis was performed using a Tescan scanning electron microscope equipped with the INCA Energy SEM Oxford Instruments energy-dispersive electron probe microanalysis system (Oxford Instruments plc, Abingdon, UK). Figure 6 presents the results of energy-dispersive X-ray spectroscopy (EDXS) study of the samples. It was established that the zinc proportion from metals amounted up to 98%. This indicated that, after laser treatment, mainly zinc oxide was present on the Cu–Zn alloy surface. During heating of the brass foil in air the oxidation of the material surface is intensified. The preferred formation of ZnO is due to a higher oxidation rate of Zn than that of copper, as well as zinc diffusion to the surface. In this case, zinc atoms penetrate through the subsurface layer of the Cu–Zn alloy.

**Figure 7:**
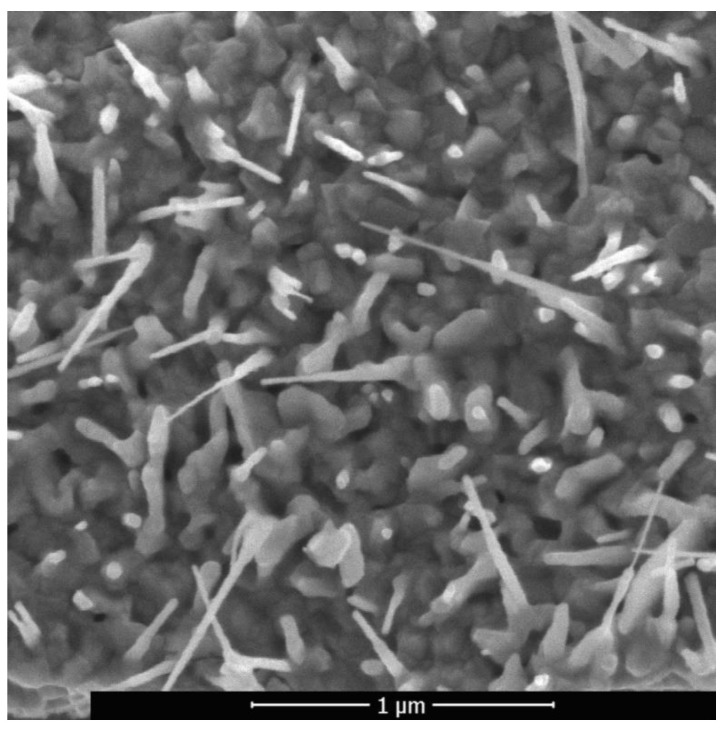
Scanning electron microscopy (SEM) image of ZnO nanowires.

The surface morphology of this selectively oxidized copper–zinc metal material was studied after laser treatment with intensification of diffusion processes in the solid state using a Quanta scanning electron microscope. It was determined that the morphology of ZnO nanostructures is sensitive both to an increase in temperature and to a local concentration of zinc due to its uneven distribution in the alloy. The intensity of arrays formation of ZnO nano-objects at the periphery was much lower than in the center since during heating the highest value of temperature was in the central area. Figure 7 shows typical ZnO nanowires formed by laser pulsed-periodic irradiation in the region with a maximum temperature of up to approximately 600 °C at a time of 23 s. Raising the temperature is an effective mean to increase the atoms mobility, since the coefficient of diffusion exponentially depend on the temperature. However, the condition for intensification of diffusion processes in the solid state of a metallic material is non-stationary local form transformation caused by sound waves induced by laser.

Nevertheless, a higher temperature favored lateral and branched growth of some ZnO crystals. In References [61,62,63], this effect during direct heating of zinc-containing materials in air was explained by the so-called self-catalytic crystal formation and subsequent crystal growth. With an increase in temperature in local areas with a high concentration of Zn, oxygen diffusion processes are intensified, which cause zinc oxidation. The formed ZnO is the core of crystallization. The deposition of ZnO clusters leads to a growth of nanowires. Their further growth, which significantly depends on temperature, is supported by the diffusion of zinc atoms to the peak. An increase in temperature leads to an increase in diffusion and a larger supply of zinc. In this case, longer nanowires are formed and there is a more intensive lateral growth in their root part. When a certain length is reached, their growth slows down due to a loss in the efficiency of supplying zinc to the peak. Therefore, the length of ZnO nanowires during the implementation of the selected laser treatment modes does not exceed 3 μm. It was shown in Reference [45] that Cu acts as an obstacle to the lateral growth of ZnO structures, i.e., when heated in air or in another oxygen-containing medium, the copper content in brass provides a natural way to control ZnO nanostructures.

**Figure 8:**
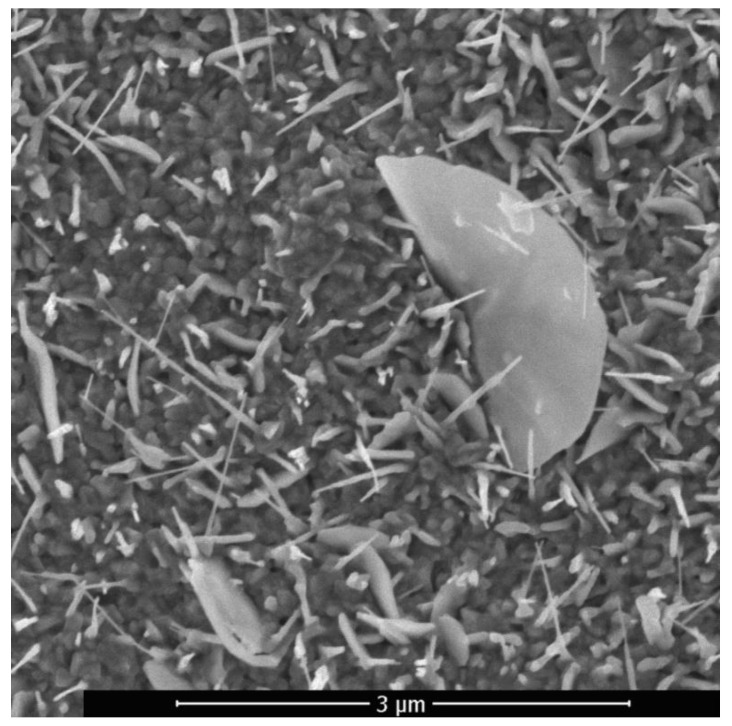
ZnO structure consisting of nanowires and nanosheets.

In the region of higher temperatures, in addition to the described branched nanostructures, so-called palmlike nanoflakes, including individual branched nanoflakes and ZnO nanosheets, were present on the samples surface. The ZnO structure, consisting of nanowires and nanosheets, was formed upon laser pulsed-periodic irradiation in the region with a maximum temperature above 600 °C at a time of 23 s is shown in Figure 8. With an increase in the treatment time and, correspondingly, temperature in the central region of the sample, exfoliation of the material was initiated. An important feature of surface atoms of the metallic material is that part of the bonds is unfilled. Therefore, surface atoms easily form bonds with atmospheric oxygen by adsorbing it. Zinc emerging from internal volumes to the surface interacts with oxygen. A loose oxide layer is formed, the elements of which are fibers with the thickness of tens of nanometers and a length of several micrometers. As the process develops, its layer density increases due to the addition of new portions of zinc from internal volumes and their oxidation. In this case, zinc is oxidized and then introduced in the layer, causing its build up.

The formed ZnO layer is highly porous and defective, due to the periphery compressive stress. Further oxidation makes the Zn atoms to move in the direction of the oxide layer/air interface by two paths: grain-boundary and lattice diffusion. In which case the lattice diffusion leads to a continuous increase of the ZnO layers, and the grain-boundary diffusion to the formation of nanowires. The dependence of the nanowire length from the distance to the center (essentially from the change in heating temperature over time) can be attributed to the Zn-ions grain-boundary diffusion to oxide layer/air interface. Changes in the nanowire transverse size during growth at various temperature regimes are small. However, the density of arrays formation of zinc oxide nanoobjects at the periphery is much lower than at the center.

The ZnO nanowires with predominantly vertical orientation that are formed in the region with a maximum temperature below 600 °C (Figure 4) have an average transverse size of about 30–40 nm and a length of 500–600 nm. The maximum length of nanowires in this region is 1 µm, the density is 10–20 µm^−2^. In the region with a maximum temperature above 600 °C nanowires alternate with nanosheets. The synthesized nanowires are also reinforced on an oxide layer and have a length of ~0.5 to 3 μm, and a diameter of ~40 to 60 nm. The longitudinal dimension of the nanosheets reaches the value of 3 μm and the transverse size of 1 μm.

**Figure 9:**
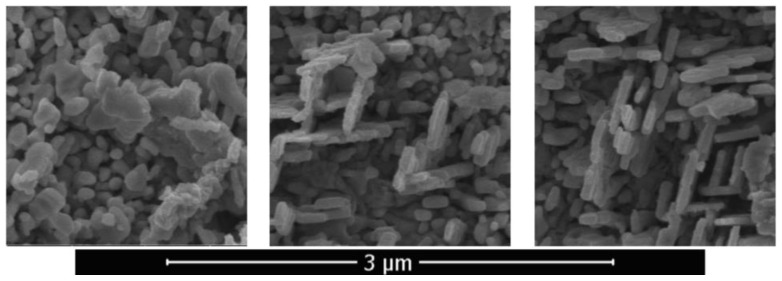
Disordered structure of the central region (on **left**), intermediate hybrid structure (in the **center**) and two-dimensional objects (on **right**).

**Figure 10:**
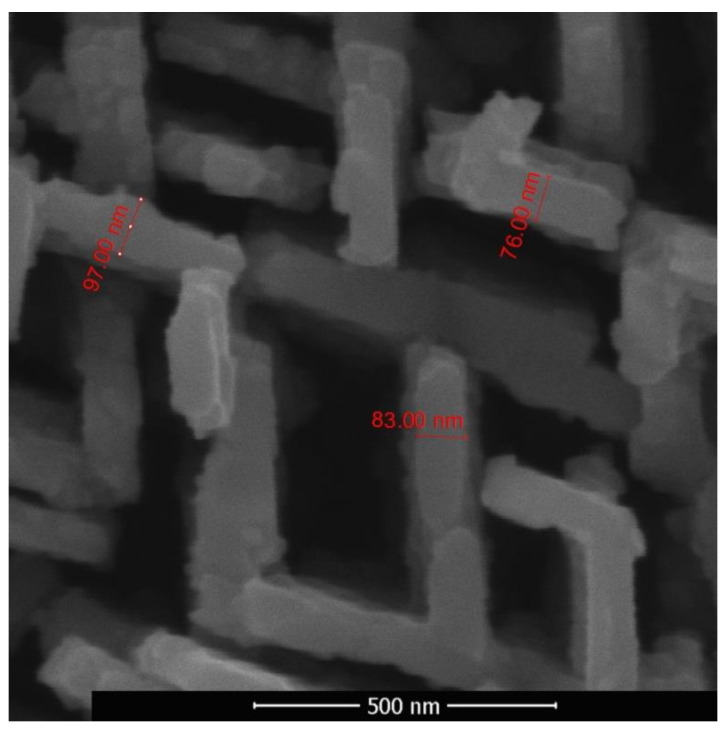
Array of two-dimensional nano-objects with characteristic thicknesses of 70–100 nm.

The thermal conductivity of oxide formed on the sample’s surface is significantly lower than that of an unoxidized metal material; therefore, the film acts as a thermal barrier coating that undergoes the greatest heat during laser treatment. Further laser irradiation leads to sintering of the particles of the formed oxide film, and strong bonds are formed between them. These processes lead to a density increase of the film, while its adhesion to the substrate surface in the sample central region, which is characterized by the most intense laser treatment with reaching higher temperatures decreases. When the value of the temperature increases to 500–600 °C (and briefly up to 800 °C) during oxidation, takes place the exfoliation of the oxide layer and this process becomes more intense with time. The thermal expansion coefficient of brass in this range is more than six times greater than that of ZnO [64,65]. During cooling process, the brass plate shrinks more than ZnO layer, due to the big difference in thermal expansion coefficient. This explains the reason for the increased exfoliation rate during high oxidation temperatures. Moreover, some cracks are observed at the bottom surface of the oxide layer which may form during cooling of brass substrate. Taking into account that exfoliation occurred when the material was cooled in air from a temperature of 500–800 °C, it can be assumed that these structures had time to form in the cooling material after laser irradiation. After exfoliation of both front and back sides of samples of the initially formed layers containing predominantly zinc oxide, probably from the zinc-depleted subsurface layer, in addition to a disordered structure, a structure was formed in the central region containing two-dimensional objects shown in Figure 9. The intermediate hybrid structure contained disordered as well as two-dimensional objects. An array of two-dimensional nano-objects with characteristic thicknesses of 70–100 nm, in local areas situated mainly in two directions and intersecting each other, is shown in Figure 10. Two-dimensional objects have nanometer size only in one dimension, and in the other two this size is macroscopic. Their quasi-two-dimensionality provides the opportunity to ensure a directional transport of charge carriers.

When compared with the material structures described in References [43,44,58], no qualitative or significant quantitative differences were found, except for the structures of the central region, formed after heating to a temperature of 700–800 °C and the delamination of the initially formed layers. This confirms the conclusions from References [66,67] which state that different laser frequency has practically no effect either on the growth rate of nanowires or on their geometric dimensions. The vibration damping in this case significantly reduces the vibration intensity values; thus, the growth rate of nanowires is also significantly reduced, probably to the values that occur during the implementation of the processes of direct heating of brass in air. Laser-induced vibrations are a condition for the intensification of nanowire growth during laser heating.

**Figure 11:**
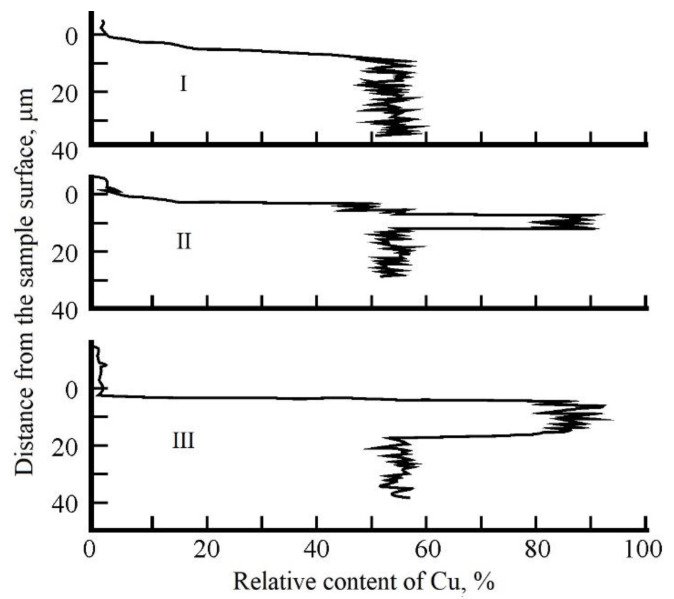
Results of studies of the chemical composition of the near-surface layer of samples regions: I—not exposed to laser irradiation; II—in the peripheral region; III—in the central region of the heat-affected zone after laser treatment.

X-ray spectral analysis was performed using an REM-100U scanning electron microscope. The results of studies of the chemical composition of the near-surface layer of samples are shown in Figure 11. The upper graph (I) shows the distribution of copper over the depth of the near-surface layer of the sample, which was not exposed to laser irradiation. The middle graph (II) shows the relative content of Cu depending on the distance to the samples surface in the peripheral region of the heat-affected zone after laser treatment, and the lower graph (III) in the central region of the heat-affected zone. A change in the chemical composition was registered, i.e., concentration of alloy components in the near-surface layer. It was established that the surface of the samples after laser treatment is a layer enriched with copper up to 90% and with a reduced zinc content up to 10%.

**Figure 12:**
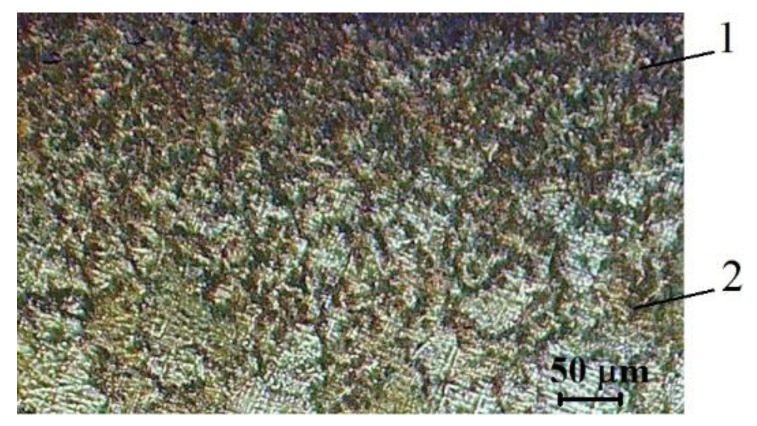
Structure of the near-surface layer of the L62 copper–zinc alloy after laser treatment. Etched section, the surface of which is located at an angle of about 5° to the treated surface. At such an angle of inclination, the size of studied section on the image increased by 11.4 times. 1—near-surface layer; 2—structure of the material without pores.

To analyze the microstructure of the subsurface layer, thin sections with a plane made at an angle to the layer under study were made. This made it possible to expand the investigated section, while at an angle of inclination of 5° to the surface, the size of this section on the image increased by 11.4 times. The microstructure was investigated in the field of view of a Neophot-30 metallographic microscope at various magnifications, both on non-etched sections and on those subjected to etching. Figure 12 shows the structure of the near-surface layer of the L62 copper–zinc alloy after laser treatment. The surface of the etched section is located at an angle of about 5° to the treated surface. To identify the features of the formation of material microstructure, a solution of the following composition was used: ferric chloride-10 g; hydrochloric acid-25 mL; and water-100 mL.

The chosen technique for metallographic studies made it possible to establish that in the near-surface layer to a depth of 15–25 µm, pores are formed, both single and forming branched channels, with a characteristic size of up to 1 µm. In the cross section, the pores are oriented from the surface into the depth of the metal. On the surface, the pore density is higher and there are also channel-type pores. In the near-surface layer, the formation of pores is predominantly along the boundaries of grains and blocks, resulting in the formation of new boundaries and, as a result, grain refinement. This structure is formed during the diffusion motion of zinc atoms to the surface. With the formation of zinc oxide on the surface of the alloy, a concentration gradient is created in the material, and subsequently zinc can be oxidized on the surface to the extent that its diffusion to this surface is ensured. A change in the colour of the near-surface layer is an indirect sign of a decrease in the zinc content in the Cu–Zn alloy of the substrate.

An assessment of the level of internal stresses in the subsurface layer of the metal material of L62 brass before and after the pulse-periodic laser irradiation was performed. To carry out an X-ray diffraction analysis, was used an DR-01 Radian X-ray diffractometer, tube type: 0.15BSV-33-Cu with beta filter of 30 μm nickel foil and CsI scintillating crystal detector. As a result of the performed analysis, it was identified that after pulse-periodic laser irradiation, the most significant changes in the intensity of diffraction maxima occur along the lines of the atomic intercrystalline plane with the interference index (311), which has a high resolution. These lines were selected for a quantitative assessment, during which a software correction of the total integral intensities of the lines by the background value was implemented. Line intensities were determined by approximating the X-ray diffraction pattern for all background points using the least squares method, respectively, the measurement interval was divided into several sections of reflection angles values, and the coefficients of the background polynomial were determined for each section while maintaining a constant degree of the polynomial. An analysis of the results of X-ray structural studies of a metallic material in the initial state and after laser treatment showed that:

1. A difference in the width and angle of the lines after laser treatment indicates a change in the density of dislocations and a redistribution of internal stresses.
2. Laser treatment in the central region of the heat-affected zone leads to an increase by 1.9 times in the intensity of the α (311) line compared to the intensity of this line for the material in the initial state; in the peripheral region of the heat-affected zone, it decreases by 1.4 times, which indicates a significant redistribution of the grain orientation in the alloy structure.

It was found that as a result of pulse-periodic laser treatment in the near-surface layer of a metal material, relative internal stresses of about 11 × 10^3^ are formed, which corresponds to the value of σ = 2.8 GPa when evaluating the main residual stresses (assuming a plane-stressed state). It has been established that during laser pulse-periodic irradiation on brass, the component of the metal alloy, namely, zinc, will oxidize on the surface in the extent that its diffusion to the surface will be ensured. The diffusion coefficient of zinc can be determined by taking into account the amount of already diffused and oxidized component of the substance. The values of the diffusant concentration on the surface and at a selected depth of the material, which were determined as a result of studies of the elemental composition were identified as boundary conditions when solving the diffusion equation. Upon direct heating and exposure to a temperature of 700 °C for 40 min, 160 min, and 360 min, the average values of the diffusion coefficient of Zn in the copper–zinc alloy in air were 9.5 × 10^−8^–1.5 × 10^−7^ cm^2^/h. During laser pulse-periodic heating under conditions of the experiment, the diffusion coefficient was 3.2 × 10^−7^ cm^2^/h; that is, 2–3 times higher than from direct heating.

The study of the electrical resistance of the created samples by the contact probe method was performed. The four-point probe method was used to determine the resistivity in the direction parallel to the plane of the material surface. In most cases, a so-called contact potential difference occurs at the point of contact between the measuring probe and the semiconductor, which influence the measurement results. In this regard, the value of the semiconductor resistance, as a rule, cannot be measured by simply connecting it to an ohmmeter circuit. Therefore, the resistivity measurement technique was applied to compensate this additional potential difference. A resistivity meter ROMETER based on the four-point probe method was used. Local specific electrical resistance of different surface areas was established at an ambient temperature of 23 °C and a relative humidity of 67%. It was determined that the specific electrical resistance at the center of the sample was 7 × 10^2^ Ohm × cm, this was 30–40% more than at the periphery. To determine the possibility of using the obtained material based on zinc oxide for the creation of sensors, oxygen was adsorbed on the sample for 5 min in an oxygen–argon mixture (70–80% oxygen, and 20–30% argon), and then the electrical resistance in the central part was measured. It was found that the adsorbed oxygen increases the electrical resistivity of the sample to 1.2 × 10^3^ Ohm × cm, i.e., by 70%. As shown in Reference [68], a change in the conductivity of zinc oxide films is associated with a change in the concentration of oxygen vacancies in the film. To improve the electrical conductivity in the literature, it is recommended to use doping, for example, with elements of the third group—boron [69], gallium [70], and aluminium [71].

It is known that the use of nanoporous oxide materials in the manufacture of chemically resistive gas sensors and the formation of sensitive layers of a gas sensor with a large specific surface area based on 1D–3D nanostructures, allows the possibility of increasing its output characteristics, in particular, to increase the sensitivity of the response to the active gas, reduce power consumption, etc., which is determined by the combination of improved physicochemical properties of nanomaterials and nanostructures used in its construction. As a rule, for known devices, a sensor layer that was formed on the basis of 1D–3D nanostructures, in addition to the reproducibility of the output parameters of the gas sensor, has the problem of forming an electrical contact between the gas-sensitive oxide layer and the metal substrate. The formation of an oxide layer directly from this substrate will solve this problem. In addition, the ZnO structure can be used to create a periodic localized electric field when a constant voltage is applied, which can modulate the optical properties of the medium in which the liquid crystals are dissolved. The created nanomaterials can find application in electrically switchable diffraction gratings that operate under the control of applied electrical energy. The main focus will be on measuring and comparing the value of local resistivity for different parts of the surface. The total resistance of a metal-semiconductor contact to direct current is defined as the sum of the contact resistance and the resistance of the oxide material. The effective dielectric constant of porous oxides can vary over a wide range with changes in properties of an environment. It is supposed to determine to what extent the electrical properties of the oxide layer can change with changes in properties of the environment: temperature and humidity. This will allow us to more fully characterize the resulting nanostructured surfaces.

## 4. Conclusions

The regularities and features of the formation of arrays of zinc oxide nano-objects with varying morphology by laser processing with intensification of diffusion processes in the solid state of Cu–Zn metallic materials which are selectively oxidizable are determined. Such arrays can provide the possibility to create a periodic localized electric field when applying a direct current, which allows the production of electrically switched diffraction gratings with a variable nature of zones.

Pulse-periodic CO_2_-laser irradiation was performed with a frequency of 100 Hz. The spectra of samples’ responses to vibrational excitation caused by a laser irradiation were studied. It was confirmed that the values of rate of vibration have local maxima in case of frequencies, which are multiples to the force fundamental frequency, values of which decrease with increasing frequency. In addition, it was established that increased values of vibration rate occur at frequencies that are near the natural oscillation frequency. In this case, laser irradiation with pulsed-periodic beam allows the formation of a stress state in samples. Maximum vibration rates at the periphery of the sample, which correspond to the laser frequency of 100 Hz as well as at multiple frequencies exceed the maximum vibration rate in the center from 15% to 120%. Laser irradiation led to an increase in temperature, and in the central region it increased more with time than at the periphery.

It was shown that during laser treatment in air using the synergy of heat exposure and vibrations induced by laser with a force fundamental frequency of 100 Hz, the brass surface of samples is mainly oxidized with the formation of zinc oxide nanowires. The intensity of arrays formation of zinc oxide nano-objects is limited by the diffusion of zinc to the surface. The condition for intensification of diffusion processes in the solid state metallic materials was caused by sound waves induced by laser. As a result of EDXS studies, the prevailing content of ZnO, and practically the absence of copper oxide in the structure of the surface layer after laser heating, were confirmed. The preferred formation of ZnO is due to a higher oxidation rate of Zn than that of copper, as well as zinc diffusion to the surface.

The formed ZnO layer is highly porous and defective, due to the periphery compressive stress. ZnO nanowires with predominantly vertical orientation that are formed in the region with a maximum temperature below 600 °C have an average transverse size of about 30–40 nm and a length of 500–600 nm. The maximum length of nanowires in this region is 1 µm, the density is 10–20 µm^−2^. In the region with a maximum temperature above 600 °C nanowires alternate with nanosheets. The synthesized nanowires are also reinforced on an oxide layer and have a length of ~0.5 to 3 μm, and a diameter of ~40 to 60 nm. The longitudinal dimension of the nanosheets reaches the value of 3 μm and the transverse size of 1 μm.

The implementation of laser treatment modes with a higher heating temperature contributed to the lateral and branched growth of some ZnO crystals, the appearance of palmlike nanoflakes and ZnO nanosheets. When initiating the processes of exfoliation of initially formed layers from both front and back sides of the samples, in addition to a disordered structure, a structure was formed in the central region containing two-dimensional objects with characteristic thicknesses of 70–100 nm. Taking into account that exfoliation occurred when the material was cooled in air from a temperature of 500–800 °C, it can be assumed that these structures had time to form in the cooling material after laser irradiation. The quasi-two-dimensionality of such nano-objects provides the opportunity to ensure directional transport of charge carriers.

Different laser frequency has practically no effect on the growth rate and geometric dimensions of nanowires. The values of vibration intensity are significantly reduced by the vibration damping and in this case the growth rate of nanowires reduces as well. This means that laser-induced vibrations are only a condition for the intensification of nanowire growth during laser heating.

Using spectral analysis, a change in the concentration of alloy components in the near-surface layer was registered. It was established that the surface of the samples after laser treatment is a layer enriched with copper and with a reduced zinc content. To analyze the microstructure of the subsurface layer, thin sections with a plane made at an angle to the layer under study were made. Results of metallographic studies made it clear that pores are formed predominantly along the boundaries of grains and blocks in the near-surface layer as result of laser treatment. The change of the near-surface layer colour is an indirect sign of a decrease in the zinc content of the Cu–Zn alloy.

Before and after the pulse-periodic laser irradiation an assessment of the level of internal stresses in the subsurface layer of the metal material of L62 brass was performed. An analysis of the results of X-ray structural studies of a metallic material in the initial state and after laser treatment showed that a change in the density of dislocations and a redistribution of internal stresses was indicated by the difference in the width and angle of the lines after laser treatment. Laser treatment lead to an increase in the intensity of the α (311) line by 1.9 times in the central region, and a decrease by 1.4 times in the peripheral region of the heat-affected zone, which indicates a significant redistribution of the grain orientation in the alloy structure. Relative internal stresses of about 11 × 10^3^ were formed in the near-surface layer of the metallic material as result of pulse-periodic laser treatment, this corresponded to the value of 2.8 GPa when evaluating the main residual stresses.

It has been established that during laser pulse-periodic irradiation on brass, the component of the metal alloy, namely, zinc, will oxidize on the surface in the extent that its diffusion to the surface will be ensured. The diffusion coefficient of zinc was determined by taking into account the amount of already diffused and oxidized component of the substance. During laser pulse-periodic heating under conditions of the experiment, the diffusion coefficient was 3.2 × 10^−7^ cm^2^/h; that is, 2–3 times higher than from direct heating and exposure to a temperature of 700 °C.

The study of the electrical resistance of the created samples by the contact probe method was performed by the four-point probe method. A resistivity meter based on the four-point probe method was used. It was determined that the specific electrical resistance at the center of the sample was 30–40% more than at the periphery. To determine the possibility of using the obtained material based on zinc oxide for the creation of sensors, oxygen was adsorbed on the sample for 5 min in an oxygen–argon mixture (70–80% oxygen, and 20–30% argon), and then the electrical resistance in the central part was measured. It was found that the adsorbed oxygen increases the electrical resistivity of the sample to 1.2 × 10^3^ Ohm × cm, i.e., by 70%. Thus, it was found that the ZnO structure can be used in chemically resistive gas sensors and electrically switchable diffraction gratings that operate under the control of applied electrical energy. The formation of an oxide layer directly from the metal substrate can solve problem of forming an electrical contact between the gas-sensitive oxide layer and this substrate.

## References

[1] Bonod N., Neauport J. (2016). Diffraction gratings: From principles to applications in high-intensity lasers. Adv. Opt. Photonics.

[2] Odinokov S.B., Shishova M.V., Zherdev A.Y., Kovalev M.S., Galkin M.L., Venediktov V.Y. (2019). Modeling of phase shifts of light in orders of diffraction gratings of an interference linear displacement sensor. Opt. Spectrosc..

[3] Kotakonda P., Naydenova I., Howard R., Martin S., Toal V. (2009). Fabrication of switchable liquid crystal devices using surface relief gratings in photopolymer. J. Mater. Sci. Mater. Electron..

[4] Choi T.H., Woo J.H., Huh J.W., Jeon B.G., Yoon T.-H. (2018). Control of haze value using electrically switchable liquid crystal phase grating devices. Proc. SPIE.

[5] Won K., Palani A., Butt H., Hands P.J.W., Rajeskharan R., Dai Q., Khan A.A., Amaratunga G.A.J., Coles H.J., Wilkinson T.D. (2013). Electrically switchable diffraction grating using a hybrid liquid crystal and carbon nanotube-based nanophotonic device. Adv. Opt. Mater..

[6] Kim U.J., Kim S.I., Hwang S., Hur J. (2014). Fabrication of two-dimensional zinc oxide nanorod patterns and their application for optical diffraction grating effect. J. Mater. Sci..

[7] Goel S., Kumar B. (2020). A review on piezo-/ferro-electric properties of morphologically diverse ZnO nanostructures. J. Alloys Compd..

[8] Borysiewicz M.A. (2019). ZnO as a functional material, a review. Crystals.

[9] Theerthagiri J., Salla S., Senthil R.A., Nithyadharseni P., Madankumar A., Arunachalam P., Maiyalagan T., Kim H.-S. (2019). A review on ZnO nanostructured materials: Energy, environmental and biological applications. Nanotechnology.

[10] Campos A.C., Paes S.C., Correa B.S., Cabrera-Pasca G.A., Costa M.S., Costa C.S., Otubo L., Carbonari A.W. (2020). Growth of long ZnO nanowires with high density on the ZnO surface for gas sensors. ACS Appl. Nano Mater..

[11] Aisida S.O., Obodo R.M., Arshad M., Mahmood I., Ahmad I., Ezema F.I., Zhao T.-K., Malik M. (2019). Irradiation-induced structural changes in ZnO nanowires. Nucl. Instrum. Methods Phys. Res. B.

[12] Al-Ruqeishi M.S., Mohiuddin T., Al-Habsi B., Al-Ruqeishi F., Al-Fahdi A., Al-Khusaibi A. (2019). Piezoelectric nanogenerator based on ZnO nanorods. Arab. J. Chem..

[13] Di Mauro A., Zimbone M., Fragalà M.E., Impellizzeri G. (2016). Synthesis of ZnO nanofibers by the electrospinning process. Mater. Sci. Semicond. Process..

[14] Hamzaoui N., Boukhachem A., Ghamnia M., Fauquet C. (2017). Investigation of some physical properties of ZnO nanofilms synthesized by micro-droplet technique. Results Phys..

[15] Bhati V.S., Hojamberdiev M., Kumar M. (2020). Enhanced sensing performance of ZnO nanostructures-based gas sensors: A review. Energy Rep..

[16] Hadis M., Ümit Ö. (2009). Zinc Oxide: Fundamentals, Materials and Device Technology.

[17] Bai Z., Xu W., Xie C. (2013). Preparation and gas-sensing property of parallel-aligned ZnO nanofibrous films. Bull. Mater. Sci..

[18] Braunovich M., Konchits V., Myshkin N. (2006). Electrical Contacts. Fundamentals, Applications and Technology.

[19] Bano N., Hussain I., Sawaf S., Alshammari A., Saleemi F. (2017). Enhancement of external quantum efficiency and quality of heterojunction white LEDs by varying the size of ZnO nanorods. Nanotechnology.

[20] Yun S., Guo T., Li Y., Gao X., Huang A., Kang L. (2020). Well-ordered vertically aligned ZnO nanorods arrays for high-performance perovskite solar cells. Mater. Res. Bull..

[21] Consonni V., Briscoe J., Kärber E., Li X., Cossuet T. (2019). ZnO nanowires for solar cells: A comprehensive review. Nanotechnology.

[22] Angub M.C.M., Vergara C.J.T., Husay H.A.F., Salvador A.A., Empizo M.J.F., Kawano K., Minami Y., Shimizu T., Sarukura N., Somintac A.S. (2018). Hydrothermal growth of vertically aligned ZnO nanorods as potential scintillator materials for radiation detectors. J. Lumin..

[23] Yang D., Qiu Y., Jiang Q., Guo Z., Song W., Xu J., Zong Y., Feng Q., Sun X. (2017). Patterned growth of ZnO nanowires on flexible substrates for enhanced performance of flexible piezoelectric nanogenerators. Appl. Phys. Lett..

[24] Skompska M., Zarȩbska K. (2014). Electrodeposition of ZnO nanorod arrays on transparent conducting substrates—A review. Electrochim. Acta.

[25] Xu S., Wang Z.L. (2011). One-dimensional ZnO nanostructures: Solution growth and functional properties. Nano Res..

[26] Soifer V.A. (2012). Computer Design of Diffractive Optics.

[27] Obreja P., Cristea D., Dinescu A., Romaniţan C. (2019). Influence of surface substrates on the properties of ZnO nanowires synthesized by hydrothermal method. Appl. Surf. Sci..

[28] Das S., Srivastava V.C. (2018). An overview of the synthesis of CuO-ZnO nanocomposite for environmental and other applications. Nanotechnol. Rev..

[29] Chaudhary S., Umar A., Bhasin K.K., Baskoutas S. (2018). Chemical sensing applications of ZnO nanomaterials. Materials.

[30] Cheng A.J., Tzeng Y., Zhou Y., Park M., Wu T.H., Shannon C., Wang D., Lee W. (2008). Thermal chemical vapor deposition growth of zinc oxide nanostructures for dye-sensitized solar cell fabrication. Appl. Phys. Lett..

[31] Chen Y., Ko H.J., Hong S.K., Yao T., Segawa Y. (2002). Morphology evolution of ZnO(0001) surface during plasma-assisted molecular-beam epitaxy. Appl. Phys. Lett..

[32] Vermenichev B.M., Lisitskiî O.L., Kumekov M.E., Kumekov S.E., Terukov E.I., Tokmoldin S.Z. (2007). Electrical properties of n-ZnO/p-CuO heterostructures. Semiconductors.

[33] Lisitski O.L., Kumekov M.E., Kumekov S.E., Terukov E.I. (2009). Thin-film polycrystalline n-ZnO/p-CuO heterojunction. Semiconductors.

[34] Wang X.D., Graugnard E., King J.S., Wang Z.L., Summers C.J. (2004). Large-scale fabrication of ordered nanobowl arrays. Nano Lett..

[35] Mårtensson T., Carlberg P., Borgström M., Montelius L., Seifert W., Samuelson L. (2004). Nanowire arrays defined by nanoimprint lithography. Nano Lett..

[36] Gates B.D., Xu Q., Stewart M., Ryan D., Willson C.G., Whitesides G.M. (2005). New approaches to nanofabrication: Molding, printing, and other techniques. Chem. Rev..

[37] Lee Y.C., Chiu C.Y. (2008). Micro-/nano-lithography based on the contact transfer of thin film and mask embedded etching. J. Micromech. Microeng..

[38] Lyanguzov N.V., Kaydashev E.M., Zakharchenko I.N., Bunina O.A. (2013). Optimization of carbothermic synthesis of zinc-oxide micro- and nanorod arrays and their morphometric parameters. Tech. Phys. Lett..

[39] Kazanskiy N.L., Murzin S.P., Osetrov Y.L., Tregub V.I. (2011). Synthesis of nanoporous structures in metallic materials under laser action. Opt. Lasers Eng..

[40] Murzin S.P. (2014). Method of composite nanomaterials synthesis under metal/oxide pulse-periodic laser treatment. Comput. Opt..

[41] Banerjee S., Myung Y., Raman S., Banerjee P. (2015). Direct Growth of Flexible and Scalable Photocathodes from α-Brass Substrates. ACS Sustain. Chem. Eng..

[42] Yuan L., Wang C., Cai R., Wang Y., Zhou G. (2013). Spontaneous ZnO nanowire formation during oxidation of Cu-Zn alloy. J. Appl. Phys..

[43] Murzin S.P., Kryuchkov A.N. (2017). Formation of ZnO/CuO heterostructure caused by laser-induced vibration action. Procedia Eng..

[44] Murzin S.P., Safin A.I., Blokhin M.V. (2019). Creation of zinc oxide based nanomaterials by repetitively pulsed laser treatment. J. Phys. Conf. Ser..

[45] Zhu Y., Sow C.H., Yu T., Zhao Q., Li P., Shen Z., Yu D., Thong J.T.-L. (2006). Co-synthesis of ZnO-CuO nanostructures by directly heating brass in air. Adv. Funct. Mater..

[46] Meshkov Y.Y., Gertsriken D.S., Mazanko V.F. (1997). Mechanism of accelerated mass transfer in metals under pulse loading. Met. Phys. Adv. Technol..

[47] Mazanko V.F., Kozlov A.V., Riasniy A.V., Prokopenko G.I., Piskun N.A. (2001). The mass transfer in metals at ultrasonic treatment. Metallofiz. Noveishie Tekhnol..

[48] Gertsriken D., Mazanko V., Qiao S., Zhang C. (2009). Diffusion characteristic of several elements in copper during an electric spark processing under a constant magnetic field. Mod. Phys. Lett. B.

[49] Chen S.Y., Wu Z.W., Liu K.X., Li X.J., Luo N., Lu G.X. (2013). Atomic diffusion behavior in Cu-Al explosive welding process. J. Appl. Phys..

[50] Pogorelov A.E., Ryaboshapka K.P., Zhuravlyov A.F. (2002). Mass transfer mechanism in real crystals by pulsed laser irradiation. J. Appl. Phys..

[51] Vasil’ev L.S. (2009). To the theory of the anomalously high diffusion rate in metals under shock action: II. Effect of shear stresses and structural and phase state of the diffusion zone on the rate of mass transfer. Phys. Met. Metallogr..

[52] Bhatt P. (2010). Maximum Marks Maximum Knowledge in Physics.

[53] Xia Q., Qu W., Li Y., Zhao J. (2018). Analysis of natural vibration frequency of different support slabs under the traffic vibration based on field measurement. Instrum. Mes. Metrol..

[54] Nakatani T., Irino T. (2004). Robust and accurate fundamental frequency estimation based on dominant harmonic components. J. Acoust. Soc. Am..

[55] Gertsriken D.S., Ignatenko A.I., Mazanko V.F., Mironova O.A., Fal’chenko Y.V., Kharchenko G.K. (2005). Determining the duration of mass transfer and the temperature of metal subjected to pulsed deformation. Phys. Met. Metallogr..

[56] Mazanko V.F., Gertzriken D.S., Bevz V.P., Mironov V.M., Mironova O.A. (2010). Mass transfer under the shock compression in metal sys-defects tems with interlayer. Metallofiz. Noveishie Tekhnol..

[57] Sarac M.F., Shimpi P., MacKey J.A., Kim D., Gao P.-X. (2010). Surface dezincification and selective oxidation induced heterogeneous semiconductor nanowire/nanofilm network junctions. Cryst. Growth Des..

[58] Murzin S.P., Prokofiev A.B., Safin A.I., Kostriukov E.E. (2018). Creation of ZnO-based nanomaterials with use synergies of the thermal action and laser-induced vibrations. J. Phys. Conf. Ser..

[59] Golley B.W. (1996). Natural frequencies of rectangular plates. Mech. Res. Commun..

[60] Kalita K., Haldar S. (2018). Natural frequencies of rectangular plate with- and without-rotary inertia. J. Inst. Eng. (India) Ser. C.

[61] Fan H.J., Scholz R., Kolb F.M., Zacharias M. (2004). Two-dimensional dendritic ZnO nanowires from oxidation of Zn microcrystals. Appl. Phys. Lett..

[62] Dang H.Y., Wang J., Fan S.S. (2003). The synthesis of metal oxide nanowires by directly heating metal samples in appropriate oxygen atmospheres. Nanotechnology.

[63] Wen X., Fang Y., Pang Q., Yang C., Wang J., Ge K., Wong K.S., Yang S.J. (2005). ZnO nanobelt arrays grown directly from and on zinc substrates: Synthesis, characterization, and applications. J. Phys. Chem. B.

[64] Shirakata S., Saeki K., Terasako T. (2002). Metalorganic molecular beam epitaxy of ZnO using DEZn and H_2_O precursors. J. Cryst. Growth.

[65] Arafat M.M., Rozali S., Haseeb A.S.M.A., Ibrahim S. (2020). Direct and catalyst-free synthesis of ZnO nanowires on brass by thermal oxidation. Nanotechnology.

[66] Murzin S.P., Shakhmatov E.V., Igolkin A.A., Musaakhunova L.F. (2015). A study of vibration characteristics and determination of the conditions of nanopores formation in metallic materials during laser action. Procedia Eng..

[67] Murzin S.P., Afanasiev S.A., Blokhin M.V. (2018). Pulse-periodic laser action to create an ordered heterogeneous structure based on copper and zinc oxides. J. Phys. Conf. Ser..

[68] Dostanko A.P., Ageev O.A., Golosov D.A., Zavadski S.M., Zamburg E.G., Vakulov D.E., Vakulov Z.E. (2014). Electrical and optical properties of zinc-oxide films deposited by the ion-beam sputtering of an oxide target. Semiconductors.

[69] Hsiao J.C., Chen C.H., Yang H.J., Wu C.L. (2013). Highly textured ZnO:B films grown by low pressure chemical vapor deposition for efficiency enhancement of heterojunction silicon-based solar cells. J. Taiwan Inst. Chem. Eng..

[70] Jung H., Kim D., Kim H. (2014). The electrical properties of low pressure chemical vapor deposition Ga doped ZnO thin films depending on chemical bonding configuration. Appl. Surf. Sci..

[71] Kim D., Yung I., Kim H. (2010). Fabrication of rough Al doped ZnO films deposited by low pressure chemical vapor deposition for high efficiency thin film solar cells. Curr. Appl. Phys..

